# Proximate Drivers of Migration and Dispersal in Wing-Monomorphic Insects

**DOI:** 10.3390/insects11010061

**Published:** 2020-01-18

**Authors:** Mark K. Asplen

**Affiliations:** Natural Sciences Department, Metropolitan State University, Saint Paul, MN 55106, USA; mark.asplen@metrostate.edu; Tel.: +1-651-793-1518

**Keywords:** behavior, flight, genetics, philopatry, syndrome, threshold, trade-off

## Abstract

Gains in our knowledge of dispersal and migration in insects have been largely limited to either wing-dimorphic species or current genetic model systems. Species belonging to these categories, however, represent only a tiny fraction of insect biodiversity, potentially making generalization problematic. In this perspective, I present three topics in which current and future research may lead to greater knowledge of these processes in wing-monomorphic insects with limited existing molecular tools. First, threshold genetic models are reviewed as testable hypotheses for the heritability of migratory traits, using the sweet potato whitefly (*Bemisia tabaci*) as a case study of a behaviorally-polymorphic migratory species lacking morphological or physiological differentiation. In addition, both adaptive and non-adaptive explanations for the empirically variable relationship between egg production and flight in wing-monomorphic insects are discussed. Finally, with respect to the largest order of insects (Hymenoptera), the role of sex determination mechanisms for haplodiploidy as a driver for natal dispersal (for inbreeding avoidance) versus philopatry (such as in local mate competition) is discussed.

## 1. Introduction

“Despite these findings, much remains to be determined about dispersal by flight and its relationship to reproductive timing in parasitoid wasps.”

“Dispersal remains poorly understood when compared to other major life history processes in terrestrial insects.”

The above quotes come from my first and most recent publications on dispersal by insects, respectively [1,2]. In fact, the term that most often comes to my mind when thinking about insect dispersal and migration is “black box”. The frustrating thing about continually writing these statements, of course, is that these processes are of paramount importance to both (1) understanding life history evolution at the fundamental level [3]; and (2) forecasting the impacts of both pest and beneficial insect species [4,5,6,7].

Despite lagging behind other areas of entomological research, advances in molecular biology, physiology, and technology have substantially increased our understanding of insect movement biology. Recently, I reviewed these [2] and concluded that these breakthroughs are largely limited to species with extreme migratory phenotypes, or those that are genetic model systems. Perhaps the best exemplars of this are insect taxa exhibiting wing dimorphism [8,9]. Such dimorphism that it prohibits or permits flight can be found in one (e.g., chalcidoid wasps engaging in strong local mate competition, staphilinid beetles, Strepsiptera [10,11,12]); or both sexes (e.g., aphids, crickets, planthoppers; reviewed by [13]). It is in species of the latter category where the strongest evidence for candidate regulatory mechanisms of insect migration can be found [14]. 

Despite the scattered, independent gains of wing dimorphism across the insect phylogeny, the overwhelming majority of pterygotes are wing-monomorphic. Detailed studies of the proximate mechanisms behind movement by flight are found in relatively few such species, including monarch butterflies (a continental-scale, transgenerational migrant [15,16]), *Drosophila melanogaster* (a genetic model system [17]), and a handful of serious pests with regard to agriculture or human health (e.g., cotton bollworm and yellow fever mosquito [18,19]). In this perspective, I will discuss three topics that may yield more general insights into dispersal and migration across the understudied multitude of flying insects: (1) migratory behavior as a threshold genetic trait; (2) the relationship between egg production and female dispersal; and (3) in the order Hymenoptera, the role sex determination mechanisms play in natal dispersal strategies.

Of all the genetic, physiological and developmental factors that can come together to generate dispersal and migratory behaviors in wing-monomorphic insects, I choose only three topics to focus on here. First, from the genetic perspective, I want to take a step back from the detailed molecular studies performed on model systems such as *Drosophila* and monarch butterflies to reintroduce generalized quantitative genetic models that likely still have strong applicability to insects (Section 2). Furthermore, while species that are wing-polymorphic and/or long-distance migrants have consistently shown clear evidence for antagonism between oogenesis and flight muscle development, there is no such clarity when the lens is turned to other insect species (Section 3). Finally, I introduce sex determination mechanism as a potential driver of natal dispersal in Hymenoptera because it brings a new proximate wrinkle into the ultimate explanation (inbreeding avoidance) that has been long assumed by dispersal ecologists (Section 4).

### Definitions

The terms “dispersal” and “migration” have varied meanings within and across biological disciplines (see [20] for a detailed discussion). Unless noted otherwise, the behavioral perspective of Asplen [2] is adopted here: (1) dispersal refers to behaviors that displace insects from their natal habitat or move them between breeding habitats; while (2) migration involves persistent and straightened-out movement in which the insect both ignores typical foraging cues and abandons its home range (after [21]).

## 2. Threshold Genetic Models: Back to the Future 

In *Drosophila melanogaster*, larval foraging behavior can be tied to a Mendelian polymorphism in the *foraging* (*for*) gene: dominant “rovers” (*for*^R^) traverse a larger search area than recessive “sitters” (*for*^S^) [22,23]. Interestingly, these genotypes appear to influence adult dispersal in both laboratory and field trials, with rover adults dispersing more often and over greater distances than sitter adults [17]. Such a simple genetic mechanism as this for insect flight, however, is likely the exception rather than the rule. For example, the combination of flight mill and genomic studies of the fall armyworm (*Helicoverpa armigera*) indicate 215 candidate genes related to long versus short distance flight [19]; this is more in line with the more general expectation that behavioral traits tend to be of polygenic inheritance [24,25]. In monarch butterflies (*Danaus plexippus*), where migration is transgenerational and direction is reversed, epigenetic effects on migratory behavior are relatively easier to test for and illustrate strong environmental effects on direction switching in re-migrants [26,27].

Recent research into the genetics and epigenetics of migration has certainly increased our understanding of the phenomenon in model systems where such tools are readily available. Unfortunately, the broader application of these discoveries is problematic, as unique life history traits (e.g., transgenerational migration in monarchs over extremely long distances) that make these species ideal for studying migration are not necessarily representative of higher taxa (e.g., Insecta, Lepidoptera, Nymphalidae). When reviewing genomic techniques in migration research on two focal species (blackcaps and monarch butterflies), Merlin and Liedvogel [27] addressed this challenge:

“Ultimately, the discovery of generalizable mechanisms, or lack thereof, will, however, require the diversification of strategically chosen migratory animal models across taxa.”

Insect genetic model systems, such as *Drosophila*, clearly provide powerful tools for uncovering the proximate mechanisms underlying complex behavioral traits. A valid question, however, concerns the degree to which these findings can be generalized to the majority of understudied insect taxa. Given the increasing use of molecular techniques throughout the biological sciences, this type of work will no doubt continue to be the primary research effort towards a better understanding of migration and dispersal in insects. To increase the opportunity for generalization, however, I also advocate a complementary approach: extensively testing the assumptions of quantitative genetics models derived from first principles. Perhaps most ideal of these are threshold genetic models for dimorphic phenotypic traits [28,29], which have been applied to migratory behaviors in birds (e.g., [30]), salmonid fishes (e.g., [31]), and insects (reviewed by [32]). One advantage of this approach is that it requires little in the way of prior genetic knowledge of focal species; instead, what is chiefly needed is a clear approach to distinguish between migratory and non-migratory phenotypes. It is here that insects are preferable models to birds, for migratory behaviors are readily measured in large numbers of individuals using straightforward laboratory techniques (e.g., tethered flight mills, free-flight chambers; [33,34,35]). 

**Figure 1:**
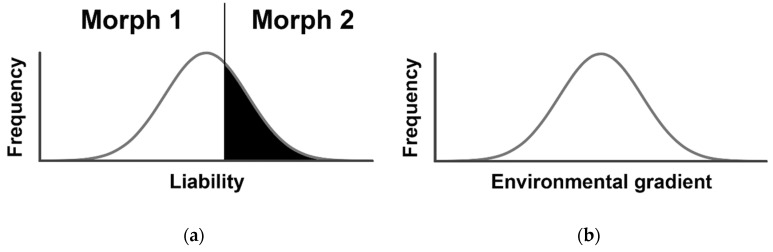
Threshold genetic models for dimorphic traits, such as migratory vs. non-migratory morphs. (**a**) The classical threshold genetic model. Liability represents the normally-distributed underlying trait (gray curve), with the vertical line indicating a fixed threshold that determines the transition from one morph to another; (**b**) The environmental threshold model. Here, the genetically-determined thresholds for transition between morphs are normally distributed (gray curve), and correspond to the level of an environmental variable. After Roff [28].

The *classical threshold genetic model* assumes that the value of a trait is determined by either the additive action of two alleles at a single locus (Mendelian inheritance) or alleles at several loci (polygenic inheritance; Figure 1a). A single, fixed threshold value of the trait determines the switch from one phenotype to another. Alternatively, the related *environmental threshold model* assumes a fixed trait with a threshold (1) that varies by genotype, (2) is normally-distributed in the population, and (3) is sensitive to some key environmental variable (Figure 1b; [36]). While quite different in their biological assumptions, Roff [28] notes that these models are mathematically equivalent as long as the underlying trait value’s mean in the classical threshold model is a monotonic function (i.e., one that is always increasing or decreasing) of the environment (see Figure 4 in [28]). Later modifications of the environmental threshold model have incorporated phenotypic plasticity in both the underlying trait value and the threshold value [30,37], although empirical support for the latter was lacking in birds.

As a parallel research initiative to molecular-based approaches, I suggest increased effort towards empirical testing of threshold genetic models in insects with subtler migratory characteristics, such as wing monomorphism and more localized distances moved. Such species are more representative of the majority of pterygotes, thus having value to achieving a more generalized understanding of insect migratory genetics. Below, I describe the biology of a model system for such research, and briefly outline an experimental approach for testing the environmental threshold model in it.

### Testing the Environmental Threshold Model

Sweet potato whiteflies (*Bemisia tabaci*) of the clade Middle East Asia Minor-1, sometimes referred to as the ‘B’-biotype or *B. argentifolii*, underwent a global invasion in the 1980s [38,39]. Hallmarks of the taxon linked to its pest status include a high degree of polyphagy (>600 host plant species), the capacity for spreading plant viruses, and a relatively high dispersal capacity for the family Aleyrodidae [38,40,41,42]. The latter of these characteristics inspired efforts to quantify the migratory capacity of *B. tabaci* in both the laboratory and field throughout the 1990s [43,44,45,46], making it perhaps one of the best studied cases of localized migration in insects [2].

The migratory capacity of *B. tabaci* has been best documented using the vertical flight chamber technique [47,48,49]. Here, individual insects are flown in a 1-m^3^ box fitted with two light sources: (1) an overhead 400-W skylight cue, which stimulates upward movement via positive phototaxis; and (2) a side-mounted green (ca. 550 nm) light cue that mimics the vegetative cues used by *B. tabaci* in foraging for host plants. A downward-oriented laminar air flow, controlled by the investigator, keeps the insect in a steady flying position. Foraging versus migratory flight behaviors are differentiated by the continued response, or lack thereof, of the insect to timed flashes of the green light cue. These studies indicate that approximately 6% of *B. tabaci* engage in flights fitting the behavioral definition of migration [48,49].

Interestingly, studies of *B. tabaci* show little or weak evidence of it possessing the morphological and/or life history traits consistent with a migratory ‘syndrome’ (*sensu* [50]). With respect to wing morphology, Byrne and Houck [51] found only subtle differences between migrant and non-migrant *B. tabaci*, while Blackmer et al. [48] unexpectedly found *smaller* wings in migratory males and no wing-dimension differences between migratory and non-migratory females. Furthermore, *B. tabaci* shows concurrent female dispersal and egg reproduction, with a positive correlation between vitellogenin levels and long-distance flight; both of these findings are inconsistent with the ‘oogenesis-flight syndrome’ sometimes seen in migratory insects (see below [46,48,49]). Taken together, the evidence suggests that *B. tabaci* exhibits a clearly detectable migratory ‘morph’ based on behavior that is largely cryptic with respect to morphology or physiological state.

The life history of *B. tabaci* is relatively simple when compared to those of more well-studied migratory insect species. Like other sternorrhynchan hemipterans, it feeds exclusively on plant phloem via stylet penetration. Eggs are inserted directly into leaf tissue, and nymphal movement is restricted to the first instar (‘crawler’) stage [38]. As a generalist feeding on many crop species, *B. tabaci* is likely not limited by host plant availability in either its endemic or invaded regions where year-round growing is common. This makes the species a strong choice for testing the assumptions of threshold genetic traits in a representative wing-monomorphic insect.

**Figure 2:**
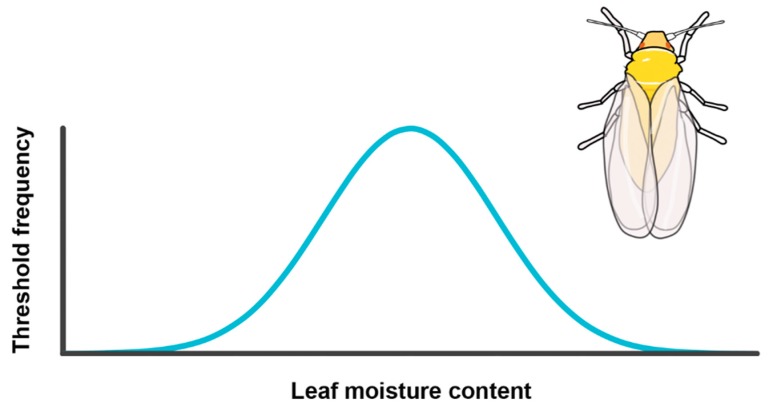
Hypothesized environmental threshold model for migration in the sweet potato whitefly, *Bemisia tabaci*. Here, the genetically-determined threshold for switching to a migratory phase (blue curve) corresponds to leaf moisture content. Whitefly illustration by Cynthia Harley.

Here, I apply the original environmental threshold model [36] as a testable first hypothesis for the genetic basis of migration in *B. tabaci* (Figure 2, Table 1). While later variants of the environmental threshold model incorporate phenotypic plasticity (see above), it is desirable to first reject the simpler alternative due to potential diminishing returns in the use of increasingly complex models. Given that (1) studies of *B. tabaci* migration were conducted on populations in southeastern Arizona, where these diurnal insects and their host plants consistently face hot, arid conditions; (2) *B. tabaci* is an exclusively fluid-feeding herbivore; and (3) scheduled defoliation is part of the standard growing practice of a major summer food source (cotton, *Gossypium hirsutum*), the environmental variable chosen to underlie migratory decisions in the model is leaf moisture content. Genetically-determined migratory thresholds are assumed to be normally distributed, with the 6% of migrants detected in previous studies representing individuals from the right tail of the distribution (Figure 2). A testable prediction of the model is that there exists a similarly low percentage of individuals that only migrate under extreme levels of plant water stress, with most individuals emigrating at intermediate levels of leaf moisture loss. 

Experiments designed to test the above model require two primary approaches: (1) establishment of the distribution of migratory responses to leaf moisture content in natural *B. tabaci* populations; and (2) strong disruptive selection for (presumably) low and high migratory thresholds in response to leaf moisture content. *B. tabaci* populations can be exposed to cotton plants of common age with differing levels of water stress, creating a gradient of leaf moisture content exposure. Behavioral assessment in the vertical flight chamber would permit estimation of changes in the ratio of migrant to non-migrant *B. tabaci* as leaf moisture content declines, which can then be compared to the assumptions of normality. Should there be relatively small frequencies of migrants on healthy leaves and non-migrants on dry leaves, respectively, then individuals of each can be bred within type over successive generations and their flight chamber behavior assessed against the leaf moisture gradient. Post-selection increases in the average frequencies of migration or philopatry in the respective populations would largely support the environmental threshold model proposed here.

## 3. The Oogenesis-Flight Relationship: It’s Complicated

Entomologists have long been interested in the relationship between flight and reproductive timing in female insects, given the high energetic costs of both egg production and investment in the flight motor. Johnson [52] proposed direct linkage between these two processes to explain a common tendency for migratory species to both (1) have a pronounced pre-reproductive period, and (2) undergo histolysis of flight muscles following settling. Termed the ‘oogenesis-flight syndrome’, it posits that physiological triggers (e.g., juvenile hormone) for migration inhibit egg production, and vice versa [52,53,54,55,56]. While still discussed as a physiological condition for migratory insects, several studies have cast doubt on its ubiquity as many species show egg production that is concurrent with (or even promoted by) migratory flight [2,55,57,58,59,60]. 

Even if flight and egg production are not necessarily linked at the hormonal level, it is reasonable to expect that such expensive traits can result in an allocation trade-off, or that high egg loads can be aerodynamically unfavorable [61]. Wing-dimorphic insects have provided the strongest evidence for oogenesis-flight antagonism; here, long-winged (LW) morphs usually show substantially reduced ovarian growth when compared to short-winged (SW) morphs [9,32]. Recently, Tigreros & Davidowitz [61] reviewed the literature on flight-fecundity trade-offs in wing-monomorphic insects. Of the 68 studies examined, 57% showed a negative association, 23% showed no association, and 13% showed a positive association between flight and fecundity. The results of this exhaustive review raise a fascinating question: what explains non-negative relationships between flight and oogenesis in insects? 

### 3.1. Alternative Adaptive Hypotheses in the Oogenesis-Flight Relationship

Assumptions of ubiquitous negative associations between flight and egg production ignore the possibility that natural selection could favor a different relationship under certain conditions. One example of this concerns the effects of time (or host) limitation on species with high early reproductive investment (measured by the ‘ovigeny index’ (OI), or proportion of lifetime fecundity available in the egg load at eclosion [62,63,64]). A trade-off between OI and adult lifespan is evident in holometabolous insects [62,65], which could lead to selective pressure for more rapid patch-leaving decisions prior to death in those species investing heavily in early reproduction at the expense of longevity [1]. For example, Stevens et al. [66] show that, in butterflies, models incorporating life history traits have far more predictive power for dispersal than wing size alone, and that different suites of life history traits are related to different dispersal elements. In terms of ovarian dynamics, for example, OI and ripe egg load have positive main effects and a negative interactive effect on flight propensity, while OI alone has a positive effect on the frequency of long distance dispersal. This implies that the classification of a ‘dispersal syndrome’ depends on the aspect(s) of dispersal being considered. 

The hypothesis that the reduced adult lifespans associated with higher OIs lead to more time-limited dispersal can be empirically tested in parasitoid wasps (Table 1). Ideally, this would be done in parasitoid taxa attacking the same host taxon, as these parasitoids presumably have similar nutrient budgets and have co-evolved against a common target. Both whiteflies and aphids possess parasitoid guilds containing both high OI and low OI taxa; the former are attacked by both *Amitus* (Platygastroidea: Platygastridae; OI ~ 1.0) and *Encarsia* (Chalcidoidea: Aphelinidae; OI < 0.5), while the latter often harbor both aphidiine braconids (OI > 0.6) and *Aphelinus* (Chalcidoidea: Aphelinidae; OI < 0.1) [62]. Comparisons of flight behaviors of such small parasitoids can be done in a vertical flight chamber [1], ideally following exposure of each tested species to different host densities and sugar subsidies (which could each potentially influence any relationship between parasitoid lifespan and patch leaving).

Time limitation via early reproductive investment is not the only potential explanatory factor for a positive relationship between flight and egg production. For example, rapid reproduction could be favored immediately following emigration from poor habitats, leading to a ‘colonizer syndrome’ of high dispersal and reproductive effort [61]. Furthermore, insects searching for ephemeral resources (e.g., plant shoots of a certain age) or that exploit patches that vary widely in quality (e.g., due to conspecific exploitation or risks of cannibalism under crowding) may be under particularly strong pressure to rapidly locate suitable patches despite considerable reproductive investment. Regardless of the specific selective pressures at play, these examples suggest that alternative hypotheses should be considered to the current null model of flight-reproduction antagonism.

### 3.2. Oogenesis and Flight May not Compete for Resources

An obvious assumption of allocation trade-offs is that two life history traits are drawing from a common resource pool. Lack of a trade-off, therefore, could indicate that different resources are being utilized for each trait [61]. For example, there is ample evidence in parasitoid wasps that carbohydrates (often obtained via nectar feeding) are the primary (or even sole) flight fuel [67,68,69]. Adult parasitoid wasps are broadly incapable of lipogenesis from ingested carbohydrates [70,71]; as such, they obtain their lifetime lipid supplies from their hosts during larval development. Lipids are critical to egg production in parasitoid wasps [67], and analysis of the complete lifetime energy budget in *Eupelmus vuilletti* suggests that carbohydrate and protein feeding by adults staves off the use of lipid reserves for somatic maintenance instead of egg production (making it simultaneously a capital and income breeder [72]). If such dynamics are the norm for nectar and host-feeding parasitoids, then tri-partite allocation relationships between flight, longevity, and reproduction would be both (1) common, and (2) not readily resolved by focusing on only two of the traits (e.g., oogenesis and flight). The interrelationships between flight fuels, nutrient allocation to reproduction, and resource acquisition strategy (capital versus income breeding) are critical areas for further study in these and other wing-monomorphic insects [61].

### 3.3. Masking of the Trade-Off between Oogenesis and Flight

Even if flight and egg production are actually competing for common resources in a wing-monomorphic species, the trade-off may not be detected empirically. The ‘Y-model’, a foundational principle in life history evolution, shows how this can occur in genetic correlations between traits [73]. Let us assume that flight and egg production linearly trade-off against each other, such that proportional investment in flight decreases investment in egg production by that same proportion. If organisms illustrate greater genetic variance in resource allocation (dispersal versus reproduction) than in resource acquisition (setting of the nutrient pool size), then a negative genetic correlation between flight and egg production will be observed. On the other hand, if there is greater genetic variance in resource acquisition than in resource allocation, a positive genetic correlation between the two traits will exist despite the underlying trade-off.

Asplen et al. [74] show how a phenotypic expression of the Y-model can explain the masking of allocation trade-offs. It builds on studies of the fig-fig wasp pollination mutualism, where the expected trade-off between seed and wasp production is only observed after variation in fig resource levels are controlled for [75]. One can easily imagine that individual insects within any species could vary widely in their ability to acquire resources via spatiotemporal variation in resource availability and/or phenotypic plasticity in traits relating to foraging success. If this approach were applied to an insect species where no oogenesis-flight relationship is found, then the flight proxy measure and egg load should be separately plotted against some measure of resource acquisition (e.g., fat body size, host size), with the residuals of each relationship then plotted against each other. If an allocation trade-off exists, the plot of residuals will have a negative slope (see Figure 1 in [74]).

## 4. Natal Dispersal and Sex Determination in Hymenoptera: Alphabet Soup

Hymenoptera likely constitutes the largest insect order, with the vast majority of this biodiversity classified as ‘micro-Hymenoptera’ and exhibiting the parasitoid life history strategy [76]. Germane to this perspective, the order can be viewed as a microcosm of Insecta itself: ancestral and predominant wing-monomorphism with isolated gains of wing-dimorphism in various lineages (see below). Unlike Insecta as a whole, however, hymenopterans are united by the synapomorphy of arrhenotokous haplodiploidy, where unfertilized eggs typically develop into males and fertilized eggs typically develop into females. Interestingly, while thought to have evolved only once, hymenopteran haplodiploidy is achieved via different sex determination mechanisms (see below). In this section, I will examine the potential role of genetic sex determination mechanisms in organizing traits related to natal dispersal (such as sexual wing dimorphism) and risks of inbreeding depression.

From the ultimate perspective, a central hypothesis for high degrees of natal dispersal by one sex is inbreeding avoidance [77,78,79,80,81]. Interestingly, many gregarious parasitoid species (i.e., those where multiple eggs are laid per host) and fig wasps exhibit such high degrees of early philopatry that mating is limited exclusively to the natal patch. This has led to the development of a foundational concept in behavioral ecology, local mate competition (LMC) [10,82]; here, natal philopatry favors the evolution of strongly female-biased sex ratios to prevent son-son conflict over mating with daughters. This effect is enhanced in haplodiploid species due to increased relatedness of mothers to daughters versus sons with inbreeding. Male winglessness is commonly observed in hymenopterans exhibiting LMC, with mated females leaving the patch solely to exploit new resources.

One of the proposed adaptive benefits of haplodiploidy is the rapid purging of deleterious, recessive traits through haploid males, which can ameliorate inbreeding depression [83]. This effect, however, is counterbalanced by strong negative effects of inbreeding in species possessing complementary sex determination (CSD) [84,85,86]. Sex under CSD is determined by allelic combinations at one (single locus CSD, or sl-CSD) or more (multi-locus CSD, or ml-CSD) autosomal genes, with heterozygotes becoming female and hemi- or homozygotes becoming male. Diploid, homozygous males almost always carry severe fitness limitations, creating a special case of severe inbreeding depression. Not surprisingly, gregarious parasitoid wasp species with CSD have been observed to readily engage in sex-biased natal dispersal, likely to avoid sib-matings [87,88].

While CSD has been confirmed in several hymenopteran species, and is likely the ancestral state in the order [89], its lack in species of several taxa (e.g., Bethylidae, Chalcidoidea, Cynipoidea) has been confirmed by experiments showing no diploid male production despite multiple generations of strong inbreeding [85,86]. In species without CSD, at least in the chalcidoid *Nasonia vitripennis*, sex determination appears to be under maternal control through a feminizing effect that is silenced in haploid offspring [90]. 

Whereas natal dispersal functions as an outbreeding mechanism in CSD species, species engaging in LMC and sex-specific flightlessness are more commonly found in taxa that apparently lack CSD (e.g., *Nasonia vitripennis* and Agaonidae within Chalcidoidea [91,92]); *Sclerodermus* within Bethylidae [93]). Ants (Formicidae) typically possess CSD [89], however, inbreeding experiments with *Cardiocondyla obscurior* revealed only a single diploid male after seven generations of inbreeding [94], supporting either ml-CSD or lack of CSD altogether (either of which would decrease diploid male production under inbreeding). Field studies of a congener, *Cardiocondyla batesii*, show strongly female-biased sex ratios and LMC, but also no evidence of diploid male production [95]. Sexual wing dimorphism with male winglessness is known in both this species and other congeners [96,97]. Taken together, these case studies suggest a role for sex determination mechanism as a driver for natal dispersal or philopatry in Hymenoptera, depending on whether or not that mechanism elevates the costs of inbreeding.

The braconid genus *Cotesia* may provide the best model system for studying the influence of sex determination mode on dispersal in Hymenoptera (Table 1). All three major modes of hymenopteran sex determination have been found in wing-monomorphic taxa within the genus: sl-CSD (*C. glomerata*), ml-CSD (*C. vestalis*), and lack of CSD (*C. flavipes*) [89,98,99,100]. Laboratory flight tests, either in the vertical flight chamber or on tethered flight mills, could be performed on *Cotesia* species of known sex determination mechanism, with the expectation that unmated individuals with mechanisms expected to produce fewer diploid males (i.e., ml-CSD or lack of CSD) should exhibit lower flight levels than those bearing the highest risk of inbreeding depression (i.e., those with sl-CSD).

## 5. Conclusions

Rather than an exhaustive review of genetic and physiological mechanisms underlying dispersal and migration in well-studied model organisms, this perspective examined relationships and hypotheses that may be more generalizable to Insecta as a whole (or, at least, to large clades within it). An important theme throughout is that, despite the theoretical attention paid to the ultimate consequences of dispersal and migratory behaviors, there is still much to learn about how they (1) are controlled genetically; (2) truly interact with other costly life history traits, such as egg production; and (3) may be driven by aspects of organismal biology that are not commonly associated with flight, such as sex determination mechanisms. The inclusion of wing-monomorphic species that are not seen as model systems for migratory/dispersal research (e.g., whiteflies, non-migratory butterflies, parasitoid wasps) will hopefully stimulate research into taxa that may be more representative of “Citizen Insect”.

## Figures and Tables

**Table 1:** Summary of major hypotheses and related tests proposed in the text.

Hypothesis	Focal Taxa	Test
ETM ^1^ explains whitefly migration, with leaf moisture content as driving factor	*Bemisia tabaci*	Vertical flight chamber studies on individuals faced with different levels of plant water stress, followed by disruptive selection
Positive relationship between OI ^2^ and flight propensity, duration and distance	Parastioids of whiteflies (*Amitus* spp.; *Encarsia* spp.) and aphids (aphidiine braconids; *Aphelinus* spp.)	Vertical flight chamber studies on individuals from representative species of each parasitoid guild with high and low OIs
Hymenopterans with sl-CSD ^3^ will exhibit higher levels of natal dispersal than those with other sex determination mechanisms	*Cotesia* spp.	Vertical flight chamber and/or tethered flight mill studies of unmated individuals from species with sl-CSD, ml-CSD ^4^ or no CSD

^1^ Environmental threshold model. ^2^ Ovigeny index. ^3^ Single-locus complementary sex determination. ^4^ Multiple-locus complementary sex determination.
